# Effectiveness of discovery learning using a mobile otoscopy simulator on knowledge acquisition and retention in medical students: a randomized controlled trial

**DOI:** 10.1186/s40463-018-0317-4

**Published:** 2018-11-20

**Authors:** Josie Xu, Paolo Campisi, Vito Forte, Brian Carrillo, Allan Vescan, Ryan Brydges

**Affiliations:** 1 Department of Otolaryngology-Head and Neck Surgery, University of Toronto, 190 Elizabeth Street 3S-438, Toronto, M5G 2C4 Canada; 2 OtoSim Inc., Toronto, Canada; 3 The Wilson Centre, University Health Network & University of Toronto, Toronto, Canada; 4 Department of Medicine, University of Toronto, Toronto, Canada; 5 Allan Water Family Simulation Centre, St. Michael’s Hospital, Toronto, Canada

**Keywords:** Simulation, Medical education, Discovery learning, Otoscopy

## Abstract

**Background:**

Portable educational technologies, like simulators, afford students the opportunity to learn independently. A key question in education, is how to pair self-regulated learning (SRL) with direct instruction. A cloud-based portable otoscopy simulator was employed to compare two curricula involving SRL. Pre-clerkship medical students used a prototype smartphone application, a 3D ear attachment and an otoscope to complete either otoscopy curriculum.

**Methods:**

Pre-clerkship medical students were recruited and randomized to two curriculum designs. The “Discovery then Instruction” group received the simulator one week before a traditional lecture, while the “Instruction then Discovery” group received it after the lecture. To assess participants’ ability to identify otoscopic pathology, we used a 100-item test at baseline, post-intervention and 2-week retention time points. Secondary outcomes included self-reported comfort, time spent using the device, and a survey on learning preferences.

**Results:**

Thirty-four students completed the study. Analysis of knowledge acquisition and retention showed improvement in scores of both groups and no significant effects of group (F_1,31_ = 0.53, *p* = 0.47). An analysis of participants’ self-reported comfort showed a significant group x test interaction (F_1,36_ = 4.61, *p* = 0.04), where only the discovery then instruction group’s comfort improved significantly. Overall device usage was low, as the discovery then instruction group spent 21.47 ± 26.28 min, while the instruction then discovery group spent 13.84 ± 18.71 min. The discovery first group’s time spent with the simulator correlated moderately with their post-test score (*r* = 0.42, *p* = 0.07). After the intervention, most participants in both groups (63–68%) stated that they would prefer the instruction then discovery sequence.

**Conclusions:**

Both curricular sequences led to improved knowledge scores with no statistically significant knowledge differences. When given minimal guidance, students engaged in discovery learning minimally. There is value in SRL in simulation education, and we plan to further improve our curricular design by considering learner behaviours identified in this study.

## Background

In Canada, undergraduate medical education curricula have gradually incorporated more opportunities for self-regulated learning (SRL) [1]. In the process, educators have explored numerous technology-assisted tools, web-based modules and simulators to supplement or replace didactic lectures and formal clinical instruction. However, the optimal way to combine traditional lectures and trainees’ SRL using simulators has yet to be studied closely in health professions education [1].

### Otoscopy education

A report published in 2008 suggests that otolaryngology is under-represented in Canadian undergraduate medical education curricula, with some schools graduating residents without any otolaryngology clinical experience [2]. In schools with otolaryngology instruction, medical students commonly attend lectures or clinical skills sessions with preceptors in otolaryngology, family medicine and paediatrics. Educators expect trainees to further refine their otolaryngology examination skills while “on the job” during clerkship and residency.

It is not surprising, therefore, that medical school graduates have reported a lack of confidence and clinical acumen when it comes to acquiring otoscopy skills [3, 4]. In an effort to enhance the quality of otoscopy instruction, several technologies have recently been developed such as a web-based otoscopy simulator [5], a rubber mannequin simulator with pneumatic otoscopy capabilities [6], and a table-top otoscopy simulator with an external ear on a digital screen projected at the base of the ear canal (OtoSim). One study has demonstrated that OtoSim improved the acquisition and retention of otoscopy skills in family medicine, pediatric and otolaryngology residents [7]. Another study showed that otoscopy simulation training was more effective than web-based modules and didactic lectures [8].

### Discovery learning

Deciding on the best curriculum design and allocation of resources requires a thorough analysis of how different instructional strategies for using simulators impacts learning outcomes [9, 10]. The concept of SRL, for example, has led to numerous interventions that have helped trainees learn on their own [11]. One intervention, discovery learning, involves giving trainees the opportunity to explore the subject matter on their own initially, before they interact with an instructor. In an ideal discovery learning condition, trainees autonomously interpret the learning task demands, experiment with different solutions to a problem, and formulate conceptual connections in a personalized way [12].

Typical discovery learning conditions in medicine include inquiry-based learning and problem-based learning [13]. Experience in such conditions can lead to ‘productive failure’, where trainees’ initial struggles to solve a problem can lead to improved retention of knowledge and skills. Discovery learning has been shown to enhance transfer of learning and increase trainees’ positive attitudes towards the learning domain [12]. However, others have argued that there is a risk of inaccurate content representation, and unnecessary trainee stress due to the added cognitive load of struggling [14, 15]. Rather than debate the isolated benefits of direct instruction and discovery learning, contemporary education scientists have started studying how to optimize the sequence or combination of the two learning approaches [12, 16, 17].

In the present study, two groups of medical students were trained with a mobile otoscopy simulator, with each group experiencing a different sequence of educational intervention. One group learned independently (discovery learning) prior to a traditional lecture. The second group attended the same lecture prior to discovery learning. We compared how well participants in the two groups acquired and retained knowledge, as well as their time spent using the simulator. We hypothesized that the ‘discovery then instruction’ group would perform better on tests of knowledge acquisition and retention.

## Methods

We designed a two-group prospective, randomized controlled study. Each arm trained with the same mobile otoscopy simulator, but followed a different sequence of direct instruction and discovery learning. The study was approved by the University of Toronto Office of Research Ethics (Protocol Reference #31021).

### Participants

First and second-year (pre-clerkship) medical students were recruited from a pool of approximately 500 students enrolled in the Faculty of Medicine, University of Toronto. The first-year students had minimal or no formal otolaryngology training experience. The second-year students had access to didactic lectures in otolaryngology as part of their undergraduate curriculum. All participants received a small-value gift certificate on study completion.

### Otoscopy simulator tool

**Fig. 1:**
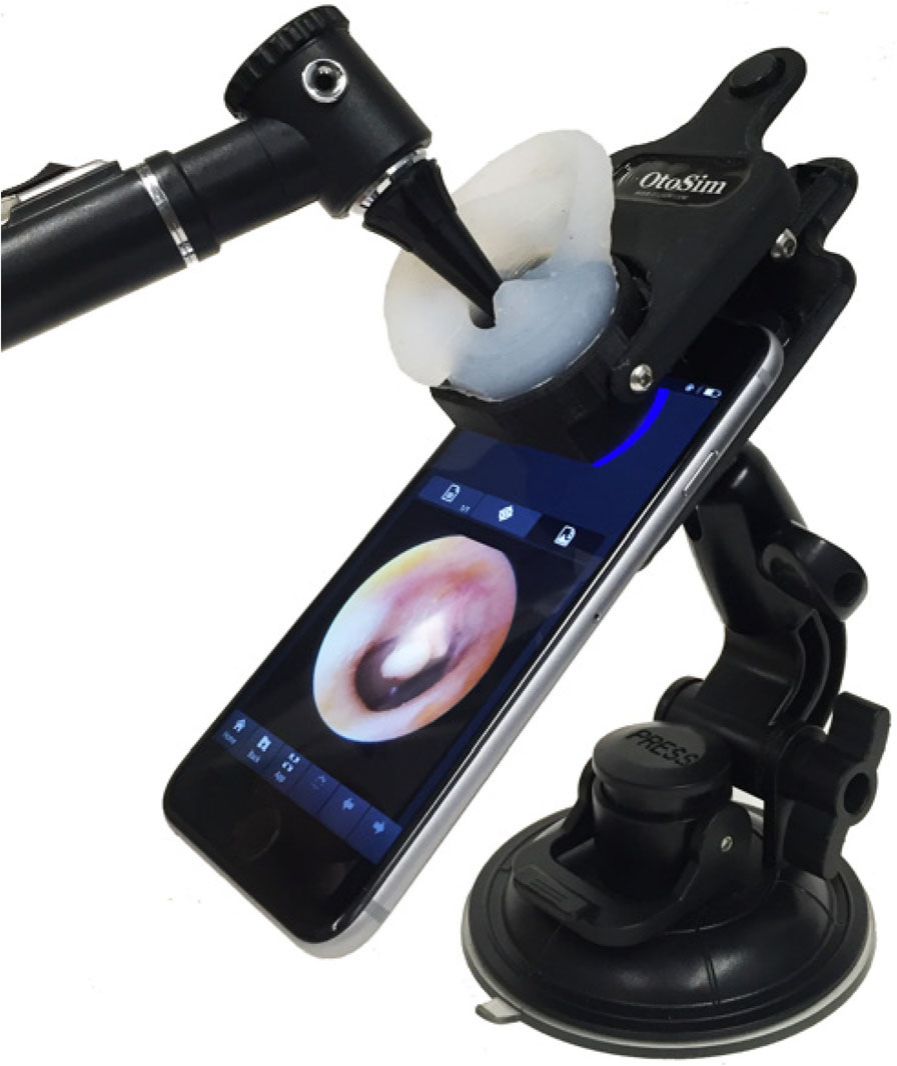
OtoSim Mobile Application, 3D Ear Attachment, Otoscope and Stand

OtoSim Mobile (OtoSim Inc., Toronto, Canada) is a cloud-based simulator that includes an online curriculum and a 3D ear attachment that connects to a smartphone (Fig. 1). Using the provided 3D ear attachment and otoscope, images were projected on the screen at the base of the ear canal to simulate otoscopy. The self-regulated curriculum included instructions on how to hold an otoscope, middle ear anatomy descriptions, a wide variety of normal and pathological middle ear images, and multiple self-assessment tools. The incorporated images were provided by the Dr. Hawke Collection.

### Didactic lecture

The first author (JX) provided an identical one-hour didactic lecture to both groups. The lecture reviewed otoscopy technique, normal canal and middle ear landmarks and common external and middle ear pathologies. The lecture also included an introduction to otoscopy simulation using desktop otoscopy simulators (OtoSim 2, OtoSim Inc., Toronto, Canada). Images were presented both on the OtoSim 2 simulation devices and projected onto classroom screens.

### Procedure

**Fig. 2:**
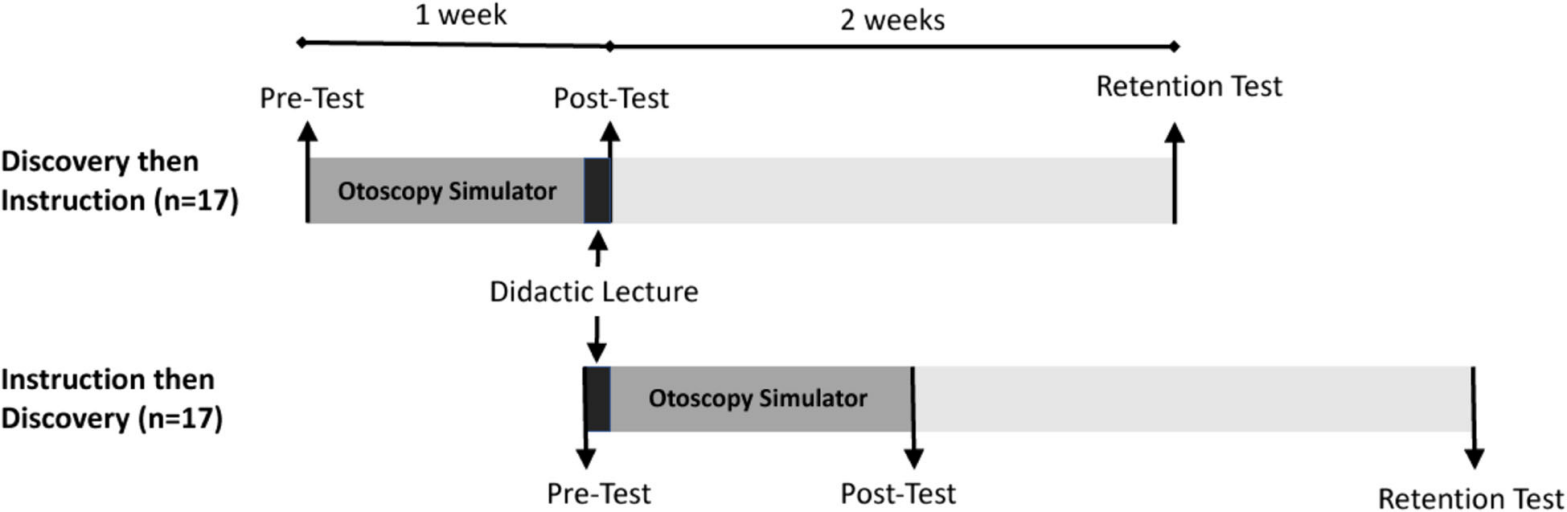
Randomized Controlled Study Design with Two Groups: (1) Discovery then Instruction, and (2) Instruction then Discovery

The full study protocol is shown schematically in Fig. 2. Forty-one students were recruited, each of whom completed baseline pretesting prior to any intervention. Participants were then assigned alphanumeric identifiers to conceal identity, stratified by year of training, then randomized to either the ‘discovery then instruction’ group (*n* = 20) or the ‘instruction then discovery’ group (*n* = 21) using an online random number generator (http://www.graphpad.com/quickcalcs/randomize2/). Neither the lecturer or researchers were blinded to participants’ group assignment.

The discovery then instruction group first received the otoscopy simulator for one week prior to their lecture. After completing the pre-test, our technicians helped set up the device on their phones, including external ear attachment, otoscope and full login access. Participants used the simulator as much as desired over one week, and did not receive instructions on which parts of the curriculum to access. After the week, they attended the one-hour lecture. We tested participants immediately after the lecture (post-test) and two weeks later (delayed retention test).

The instruction then discovery group attended the lecture immediately following their pre-test. After the lecture, participants received the mobile otoscopy simulator, which was setup appropriately on their phones. After one week with the device, they returned for an immediate post-testing. Two weeks after the post-test, they returned for the delayed retention test.

### Outcome measure

The primary study outcomes were participants’ baseline, post-intervention and retention test knowledge scores. Participants also self-reported their pre-intervention, and post-intervention comfort levels with otoscopy on a 5-point Likert scale (1 - uncomfortable, 2-slightly uncomfortable, 3 – comfortable, 4 – very comfortable, 5-expert). The participants’ time spent using the mobile otoscopy simulator during their respective discovery phases was tracked using built-in analytics. For the last set of outcomes, a pre-intervention survey was employed to document participants’ age, year of training, prior experience with otolaryngology, and learning preferences; as well as a post-intervention survey to document their scoring (5-pt Likert scale) of the simulator’s effectiveness, and their preferences for the study learning conditions. The surveys are listed in Appendix A.

**Fig. 3:**
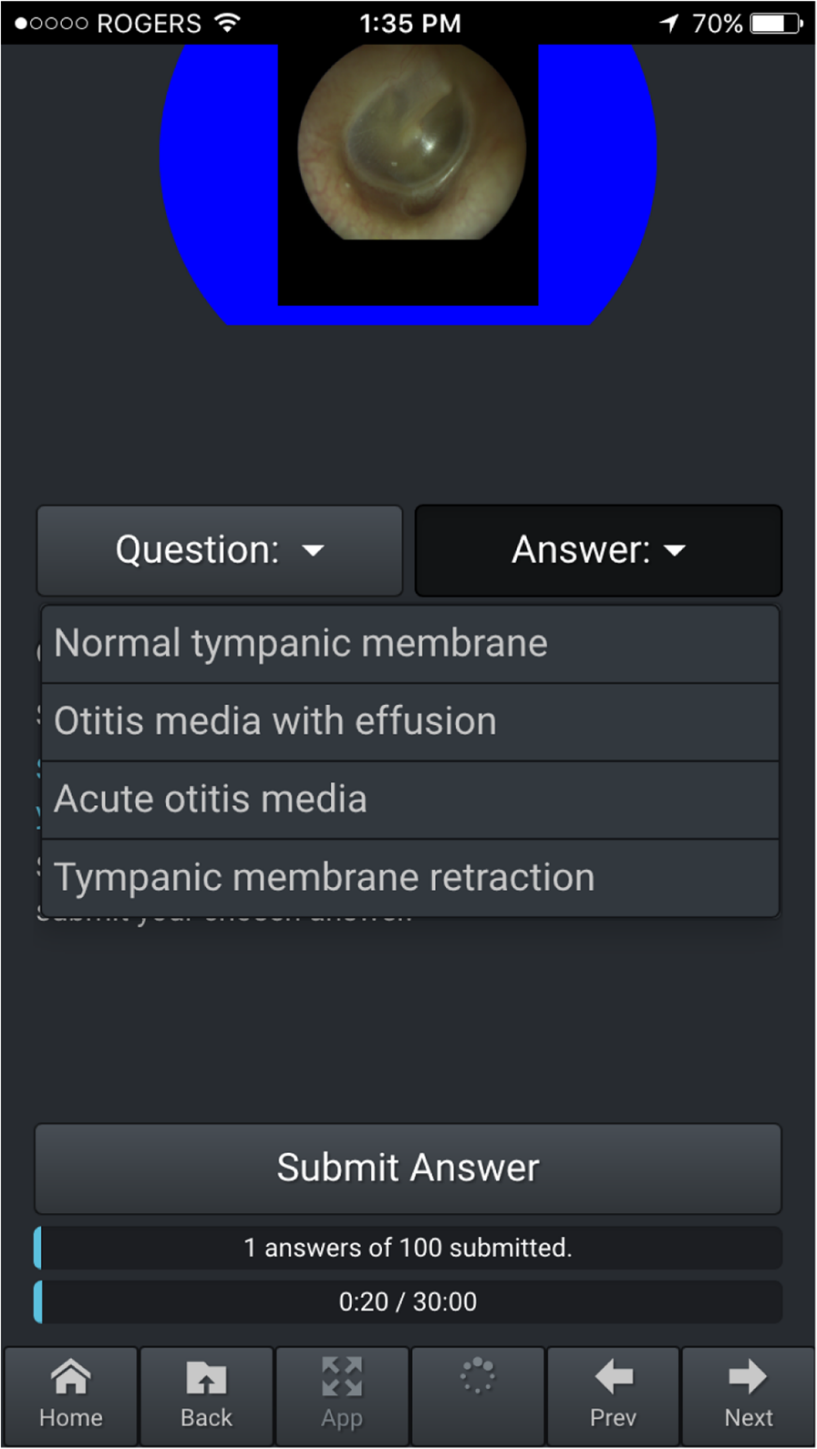
OtoSim Mobile Knowledge Test Interface

To measure participants’ knowledge, a bank of otoscopic images on the mobile otoscopy simulator was prepared. The images included a variety of normal tympanic membranes, external auditory canal pathologies and tympanic membrane pathologies. None were the same as the images on the mobile otoscopy simulator application. The test was taken on the device, and scores stored in the cloud (Fig. 3). The pre-test, post-test and retention test used the same questions, in the same order. We did not provide participants feedback or answers until after the retention test.

The first author (JX), who routinely performs otoscopy in an adult and pediatric practice, developed the knowledge test. The test included two questions based on each of 50 otoscopic images: firstly, “Is this normal or abnormal?” and secondly “What is the most likely pathology?” with four multiple choice answers. We evaluated initial drafts of the test to determine whether it could discriminate between known groups based on previous clinical experience. On an initial test of 60 images (i.e., 120 points), a staff physician identified seven images as clinically equivocal between multiple diagnoses. These images were omitted. That test showed score differentiation between a staff physician scoring 100% (106/106), a third-year resident scoring 91% (97/106), one third year medical student scoring 79% (84/106) and one second year medical student scoring 76% (81/106). Six questions were omitted and the test re-administered to three first year medical students, who scored 65% (65/100), 67% (67/100) and 70% (70/100). These pilot data were considered as providing minimal, favourable validity evidence for this knowledge test [18].

### Statistical analysis

Descriptive statistics for participants’ demographic and previous training data were calculated.

As a primary analysis, participants’ knowledge scores were examined using a 2 × 2 mixed effects analysis of covariance (ANCOVA) with group as the between-subjects factor, test (post-test, retention) as the within-subjects factor, and pre-test scores as the covariate. An ANCOVA was used to account for any variation in the post-test and retention test means arising from variation in participants’ baseline knowledge [19]. That is, the two group’s mean post-test and retention test scores were adjusted using the pre-test scores (discovery then instruction group mean: 63.24 ± 10.18, and instruction then discovery group mean: 69.41 ± 10.08).

Participants’ self-reported comfort in otoscopy was analyzed using a 2 × 2 mixed effects analysis of variance (ANOVA), with group as the between subjects factor, and test (pre-test, post-test) as the within-subjects factor. This analysis assessed the change in participants’ comfort levels.

For participants’ time spent with the simulator during their respective discovery phases, an independent samples *t*-test was conducted. For their responses regarding the preferred learning conditions post-intervention (i.e., discovery first, discovery second, either sequence), a chi-square was conducted to compare the percentage of participants responding to each option across groups.

## Results

**Fig. 4:**
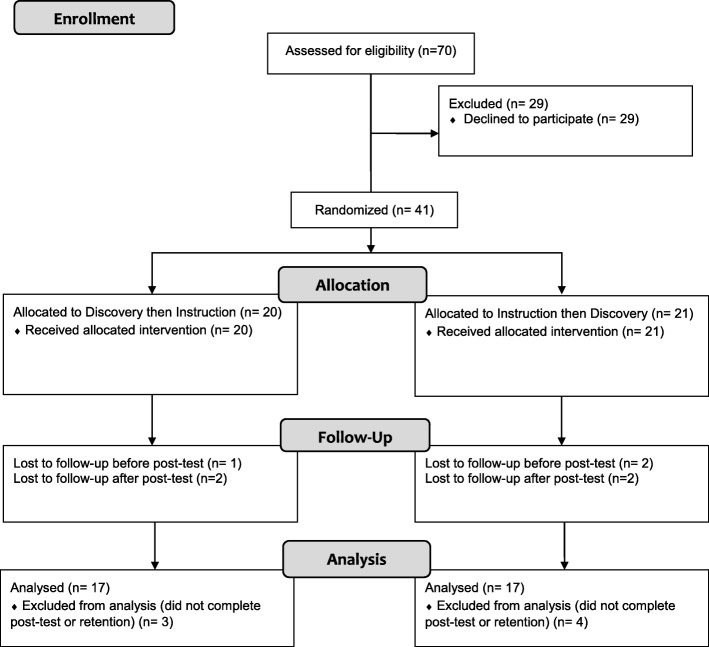
CONSORT Randomization Flow Chart

A total of 34 students completed all assigned interventions and tests. We excluded seven participants due to incomplete data (Fig. 4). The demographics of each group are shown in Table 1. We found no statistically significant difference in test scores between first and second year students.

**Table 1:** Demographic Data and Pre-Intervention Survey Results

	Discovery then Instruction (*N* = 19)	Instruction then Discovery (*N* = 19)
Mean age **±** standard deviation	23.95 ± 1.64	24.00 ± 2.03
No. 1st year medical school	*N* = 9	*N* = 7
No. 2nd year medical school	*N* = 10	*N* = 12
No. with prior experience using OtoSim	*N* = 8	*N* = 13
No. with prior ENT exposure	*N* = 16	*N* = 16
Preferred learning modality		
No. preferring Didactic lectures	*N* = 3	*N* = 3
No. preferring Small tutorials with facilitators	*N* = 11	*N* = 10
No. preferring SRL with readings	*N* = 3	*N* = 0
No. preferring SRL with modules	*N* = 2	*N* = 6

An analysis of participants’ knowledge test scores (Table 2) showed no significant effects of test (F_1,31_ = 0.06, *p* = 0.80), suggesting the groups’ maintained their knowledge over the 2-week delay. The analysis also showed no significant effects of group (F_1,31_ = 0.53, *p* = 0.47), and no significant group x test interaction (F_1,31_ = 2.46, *p* = 0.13).

**Table 2:** Participants’ Knowledge Test Scores and Post-Intervention Preferred Learning Sequence

	Discovery then Instruction	Instruction then Discovery
Knowledge Test Score		
Adjusted post-test (*N* = 17)	76.50 ± 5.28	73.33 ± 5.28
Adjusted retention test (*N* = 17)	75.23 ± 5.53	75.06 ± 5.53
Preferred Learning Sequence		
Discovery than Instruction sequence (%)	*N* = 2 (10.5)	*N* = 1 (5.3)
Instruction then Discovery sequence (%)	*N* = 12 (63.2)	*N* = 13 (68.4)
Both before and after lecture (%)	*N* = 5 (26.3)	*N* = 5 (26.3)

An analysis of participants’ self-reported comfort showed a significant effect of test (F_1,36_ = 41.47, *p* < 0.001), no effect of group (F_1,36_ = 0.50, *p* = 0.49), and a significant group x test interaction (F_1,36_ = 4.61, *p* = 0.04). Post-hoc analysis of the interaction (critical value = 0.48) revealed that the discovery then instruction group’s comfort improved significantly from pre-test (1.85 ± 0.67) to post-test (2.68 ± 0.48), whereas the instruction then discovery group’s comfort did not improve significantly (pre-test: 2.15 ± 0.37, post-test: 2.58 ± 0.61).

The discovery then instruction group spent 21.47 ± 26.28 min on the simulator, ranging from 0 to 105 min. By contrast, the instruction then discovery group spent 13.84 ± 18.71 min with a range of 0 to 73 min. These mean usage times did not differ statistically (t_36_ = 1.03, *p* = 0.31). We did find that the discovery then instruction group’s time spent with the simulator correlated moderately with their post-test score (*r* = 0.42, *p* = 0.07), but not their retention test score (*r* = 0.17, *p* = 0.51). By contrast, the instruction then discovery group’s time spent did not correlate for either the post-test score (*r* = 0.01, *p* = 0.97), or retention test score (*r* = − 0.17, p = 0.51). Relatedly, all participants in the two groups (discovery then instruction: 3.95 ± 0.52, and instruction then discovery: 3.68 ± 0.82) ranked the effectiveness of the simulator well. Most participants in both groups (63–68%) preferred the instruction then discovery sequence (Table 2); the percentage of participants favouring that sequence did not differ between the groups (χ^2^ = 0.37, *p* = 0.83).

## Discussion

We compared the effectiveness of two sequences of didactic and self-regulated, discovery learning in otoscopy simulation. Based on previous literature, we expected those starting with discovery learning would have superior knowledge retention results, however, we found no significant differences between the groups. The discovery then instruction group did experience a significant improvement in their self-reported comfort, whereas the instruction then discovery group did not. As expected, participants in the discovery then instruction group used the simulator for more time, yet did not prefer their own learning condition; all despite gaining equivalent knowledge, more comfort, and investing more time using the simulator than their peers. Below, we integrate these findings with other studies in education psychology, and consider the implications for researchers and educators in the health professions.

Our primary finding, of no significant group differences in participants’ knowledge acquisition or retention does not align with previous literature, which describes improved performance in those who experience the discovery then instruction sequence [20, 21]. We hypothesized that those exposed to discovery learning first would experience productive failure, given they were purposefully challenged to use their critical thinking skills and pre-existing knowledge to address the learning task. Previous research theorized that this difficult process of acquiring new information and reformatting existing knowledge prepares the learner for the problem-solving needed in a test or real clinical situation [22]. There are at least three potential explanations for our findings. Firstly, the discovery learning component was delivered as an informal, non-scheduled experience, meaning we asked participants to learn on their own time. Our data shows that the participants did not utilize that time well, spending only 13–22 min on average over a full week with the simulator. This lack of time investment likely limited the learning benefits of both conditions. Secondly, we created our knowledge test for this study, and the presently weak validity evidence may suggest it is not yet sensitive enough to detect the expected group differences. Thirdly, the concept of sequencing discovery learning before instruction has been associated most with measures of how well participants transfer their learning to new skills or related problems, whereas we chose to focus on assessing knowledge retention, to avoid creating multiple new assessment tools.

Despite the absence of meaningful knowledge differences, the discovery then instruction group did experience a significant increase in their self-reported comfort, invested more of their time, and yet still preferred the alternative training sequence. Not surprisingly, these busy students preferred to be taught information by an expert instead of spending time struggling to learn independently, perhaps because learners prefer fluency (i.e., perceiving the information they are learning as easy to process) over struggle [23]. However, despite their strong preferences, the instruction then discovery group did not achieve superior knowledge gains. Additional research could help understand if participants in the discovery then instruction group preferred the opposite sequence because of the difficulty they experienced with productive failure.

Both learning sequences resulted in significant knowledge improvement from pre-test to post-test, and sustained knowledge on retention test. Our findings add to the growing evidence that otoscopy simulation training improves otoscopic diagnostic accuracy [7, 8, 24, 25]. We also found that the mobile otoscopy simulator was well-received by participants.

### Study limitations

Our study has some limitations. Firstly, by using a test for the first time, we could not conduct a sample size calculation, and we suggest the study is likely underpowered. Using the adjusted mean scores for the post-test (i.e., from the ANCOVA model), a post-hoc power calculation suggests at least 44 participants per group to adequately power future studies. Secondly, the knowledge test also has weak validity evidence, in the form of discriminating between known levels of expertise, which is necessary but definitely not sufficient in the validation process [24]. One potential modification to the test would be to include more challenging questions. Thirdly, individuals spent 0–105 min practicing during the one week they had access to the simulator. Given the full simulator curriculum has been designed to address a wide range of learners, from undergraduates to senior residents, the curriculum is vast and would take over 30 h to complete. Clearly, the participants did not capitalize on the content. We purposefully studied how participants engaged in autonomous, informal learning, with the goal of mimicking realistic learning environments. Participants’ motivation for using the mobile otoscopy simulator may have been diminished after the lecture in the instruction then discovery group, thus resulting in lower usage patterns. Additionally, the study was conducted during the week before students’ final examination period, which likely affected their time allocation. We suggest that future studies seek a balance between allowing open-ended discovery learning and implementing more explicit supervision during the discovery learning period [8]. We also note the need to understand if participants’ low usage of the simulator in this study contributed at all to their knowledge beyond what they acquired by attending the lecture.

### Research implications

We are not aware of any standardized outcome measures for testing otoscopy diagnostic accuracy, which has led to a pattern in previous studies, including ours, of using experts to develop appropriate diagnoses and to select quiz images [8]. We suggest future research could focus on creating and collecting validity evidence for robust assessments of otoscopic diagnostic accuracy and clinical performance.

We also recognize the limitations of focusing on knowledge retention, rather than knowledge transfer. Hence, the proposed novel assessment tools might focus on measuring knowledge and skill transfer. For example, Wu et al. studied the efficacy of otoscopy simulator over classroom instruction and web modules in diagnostic accuracy and otoscopy skills by assessing transfer of skills to caring for real otolaryngology patients [26].

Lastly, we suggest researchers continue to seek solutions for implementing discovery learning techniques in authentic and controlled training conditions. Evidence is needed to help educators determine how to schedule an effective mix of formal and informal discovery learning opportunities.

### Clinical implications

We suggest otolaryngology educators can infer that using both didactic and simulation teaching techniques can be useful in otoscopy education, while deciding how to combine the two requires further refinement for curricular implementation. Our data showing low simulator usage suggest it may be helpful to include some form of guidance during discovery learning. Guidance could manifest as well-defined, explicit objectives, a longer training session for students to highlight key functions within the program, or discovery learning with an expert available. Our study also demonstrates the importance of seeking and incorporating student feedback and overall course load into discovery learning curricular design. Given the high amount of student motivation required for successful implementation, adding more educational modules without guidance will likely lead to lower than expected usage.

## Conclusion

Mobile otoscopy simulators can be used for SRL, and as an adjunct to traditional otoscopy education. While we did not clarify which sequence of learning results in the best acquisition, retention or transfer of knowledge, we did identify self-reported comfort, simulator usage time, and students’ learning preferences as key secondary variables to consider in future curricular design.

## Abbreviations

ANCOVA: Analysis of covariance
ANOVA: Analysis of variance
SRL: Self-regulated learning
